# Butterflies of the Bodoquena Plateau in Brazil (Lepidoptera, Papilionoidea)

**DOI:** 10.3897/zookeys.546.6138

**Published:** 2015-12-16

**Authors:** Paulo Ricardo Barbosa de Souza, Rhainer Guillermo-Ferreira

**Affiliations:** 1 Programa de Pós-graduação em Entomologia e Conservação da Biodiversidade – PPGECB, Universidade Federal da Grande Dourados – UFGD, Rod. Dourados-Itahum Km 12, Cidade Universitária, CEP 79804-970, Dourados, MS, Brazil; 2 Departamento de Hidrobiologia, Universidade Federal de São Carlos – UFSCar, São Carlos, SP, Brazil

**Keywords:** Biodiversity inventory, conservation unit, Atlantic Forest

## Abstract

Butterflies and moths are found in all terrestrial environments and require efforts for a better understanding of its mega-diversity. These taxa have been the subject of several studies involving phylogeny, ecology and environmental impacts. Nevertheless, several areas in the tropics remain unexplored, resulting in gaps in the taxonomic composition and distribution of butterflies in endemic environments. Therefore, a survey of the butterfly fauna of the Bodoquena Plateau in Brazil was conducted. This area consists of tropical Atlantic Forests, with marginal influences of Savannah, Chaco and Pantanal. Sampling was carried out in 20 locations using Van Someren Rydon traps and insect nets between November 2009 and April 2015. Active collection of individuals was conducted from 9:00 to 17:00h, totaling 240 hours of sampling effort. In total, we registered 768 individuals belonging to 146 species of 98 genera, six families and 18 subfamilies. Nymphalidae was the richest family (84 species), followed by Hesperiidae (22 species), Riodinidae (14 species), Pieridae (12) Papilionidae (11 species) and Lycaenidae (five species). We sampled 239 nymphalids in traps, with 48 species, 30 genera, 15 tribes and five subfamilies. The most common species were *Eunica macris* (Godart, 1824), *Dynamine artemisia* (Fabricius, 1793) and *Memphis moruus* (Fabricius, 1775). Therefore, this study contributes to the knowledge of the Neotropical butterfly diversity and distribution, providing 37 new records and supporting the use of wildlife inventories as important tools for the knowledge of tropical forests biodiversity and conservation.

## Introduction

Insects occupy a prominent position in biological studies on communities and habitats conservation due to its biodiversity and role in ecological processes (Elton 1973; Janzen 1987; Hölldobler and Wilson 1990; Gaston 1991; Wolda 1992, Groombridge 1992; Kato et al. 1995). Nevertheless, while insects are the most diverse group on the planet, accounting for more than half of the described living organisms, knowledge is still relatively scarce when compared to other groups (Teston and Corseuil 2002).

Butterflies and moths are found in all terrestrial environments and require efforts to better understand its mega-diversity (De Vries 1987). These taxa have been the subject of several studies involving phylogeny, ecology and environmental impacts (Brown Jr. 1996). Furthermore, the group predictably responds to environmental changes because of its microhabitat fidelity, thus facilitating rapid reactions to habitat degradation (Brown Jr. 1996). However, the natural history of most groups is still unknown, what limits conservation acts, since species respond individually to the effects of fragmentation and habitat loss (Summerville et al. 2001).

In Brazil, foreigners made the first studies on butterflies, and the first Brazilian to conduct studies was Adolpho Mabilde, who also was the first to put together a collection of Lepidoptera (Freitas and Marini-Filho 2011). Studies on butterflies were then concentrated in areas of Atlantic and Amazonian forests (Brown Jr. 1996; Uehara-Prado et al. 2004, 2009; Brown Jr. and Freitas 1999), with a few studies concentrating in the areas of Cerrado and semi-deciduous forests (Carneiro et al. 2008). For instance, there are few studies on the biodiversity of the Mato Grosso do Sul State (MS), which exhibits a set of unique endemic environments, such as the Pantanal, the Chaco and the Montaine forests of the Bodoquena Plateau.

The first studies to provide information about the butterfly fauna of MS were by Talbot (1928) and Travassos and Freitas (1941). Brown Jr. (1986) listed more than 1,000 species in a study conducted in the Pantanal region. After this study, others were carried out by Aoki and Sigrist (2006), Boff et al. (2008), Rech et al. (2009), Uehara-Prado (2009), Dolibaina et al. (2010), Aoki et al. (2012) and Bogiani et al. (2012), summing a total number of 291 species for the state. Furthermore, although this region is a priority area for studies of lepidopteran biodiversity (Freitas and Marini-Filho 2011), a large area is still unexplored.

Therefore, this study aimed to assess the diversity of butterflies of the Bodoquena Plateau, which is a conservation priority hotspot with great geological and biogeographical importance, but with insufficient data. The Bodoquena Mountains are part of the ecological corridor of Cerrado-Pantanal biodiversity, belonging to the core area of the endangered Atlantic Forest Reserve and the Pantanal Biospheres. This region has been highly threatened by tourist development and the increasing growth of agricultural practices in adjacent farms (Brazil 2007). The knowledge on the fauna of this region is scarce, except for frogs (Uetanabaro et al. 2007), macroinvertebrates (Escarpinati et al. 2011, 2013; Schulz et al. 2012), ants and wasps (Auko & Silvestre 2013; Silvestre et al. 2012; Silvestre et al. 2014).

## Material and methods

### Study area

**Figure 1.**
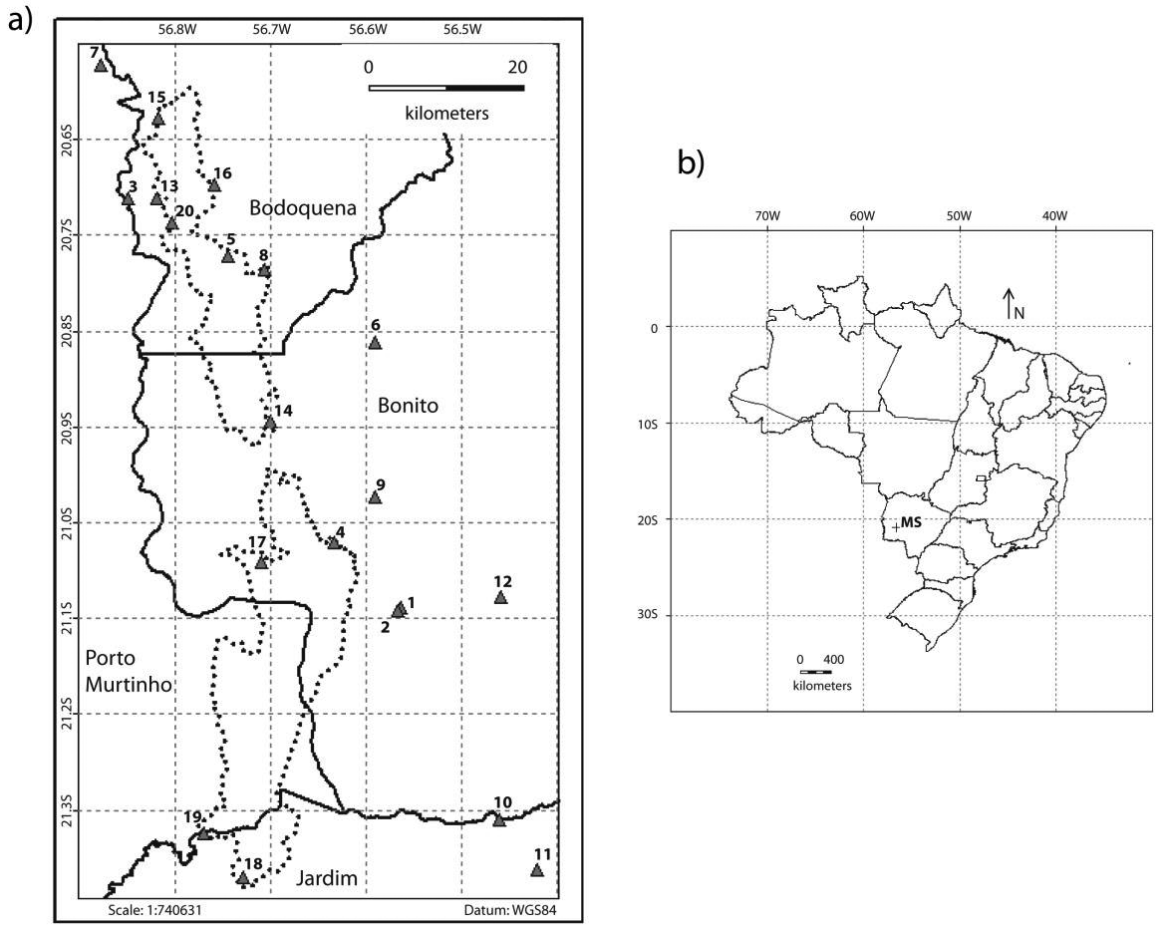
Sampling sites. Maps of the location of sampling sites in the Bodoquena Mountains in the Serra da Bodoquena National Park (**a**), and the location of the Bodoquena Plateau in Brazil (**b**).

The Serra da Bodoquena National Park (Parque Nacional da Serra da Bodoquena - PNSB) is the only conservation unit in the Mato Grosso do Sul State, located in central Brazil (21°8'2" to 2°38'26"S and 56°48'31" 56°44'28"W) (MMA 2002). It consists of two major geomorphological blocks with different characteristics: one to the north, with an area of 27.793 ha, and another to the south, with 48.688 ha (Figure 1) (Fundação Neotrópica 2002). This conservation unit has 300 km in length and width ranging from 20 to 50 km, and exhibits limestone rocks of the Corumbá Formation (Neoproterozoic III), with altitudes ranging from 450 to 800 meters (PCBAP 1997; Boggiani et al. 2000).

The vegetation is a mix of alluvial semi-deciduous forest (gallery forest), submontane deciduous forest (dry forest), wetlands, pasture and regenerating areas (Françoso et al. 2011). The predominant vegetation type is submontane deciduous forest. With an area of 764,81 km^2^, the NBSP covers approximately 0.2% of the surface of MS, which corresponds to 16% of all Atlantic Forest remnants in the state. When considering submontane deciduous seasonal forests alone, more than 25% of its area is located in this protected ecological reserve (Brazil 2007).

### Sampling procedures

Sampling was carried out in 20 locations (Table 1) using Van Someren Rydon traps and insect nets between November 2009 and April 2015. Individuals were collected with an insect net, following pre-existing trails along each locality from 9:00 to 17:00 h, totaling 240 hours of sampling effort. Each trail was sampled for 4 hours (~15-20 km), following a zigzag path along the trail. This method allowed us to sample individuals inside the forest, since we sampled along 5 m of each side of the trail. We also used bait traps with fermented banana and sugar cane juice randomly arranged at a height of two meters, with ten traps per location, totaling 200 traps. Traps were set at 10:00 h and removed eight days later at the same period of the day. Voucher specimens are deposited in the Lepidoptera Collection of the Biodiversity Museum (MuBio) of the Federal University of Grande Dourados (UFGD).

**Table 1.** Sampling sites in the Bodoquena Plateau, Mato Grosso do Sul State, Brazil.

Code	Sites	Geographic Coordinates	Height (m)	Sampling Date
1	Palmeirinhas II	21°11'5.57"S, 56°33'25.25"W	341	November 2009
2	Palmeirinhas I	21°11'16.01"S, 56°33'39.71"W	350	November 2009
3	Faz. California	20°42'5.17"S, 56°52'50.27"W	733	November 2009
4	Taquaral	21°06´27"S, 56°38´14"W	569	November 2009
5	As. Canaã	20°46'5.96"S, 56°45'43.09"W	214	November 2009
6	Faz. Pitangueiras	21°52'14’’S, 56° 35'19"W	469	November 2009
7	Kadwéu	20°32'41’’S, 56°54'44’’W	519	November 2009
8	Afluente Salobra	20°47'3.90"S, 56°43'7.37"W	447	November 2009
9	Faz. Morro Alto II	21°01'85.6"S, 56°37'47.6"W	528	November2009
10	Rio da Prata	21°25'58.80"S, 56°26'31.34"W	255	March 2011
11	Buraco das Araras	21°29'37.2"S, 56°23'52.2"W	318	March 2011
12	Hotel Cabanas	21°10'15.44"S, 56°26'24.2"W	276	March 2011
13	Nascente do Gruta	20°42'6.72"S, 56°50'43.79"W	476	March 2011
14	Marambaia	20°57'53.60"S, 56°42'43.90"W	665	December 2013
15	Faz. Sol de Maio	20°36'18.00"S, 56°50'36.40"W	399	February 2013
16	Faz. Rancho Branco	20°41'6.20"S, 56°46'43.70"W	178	December 2013
17	Boqueirão	21°7'51.30"S, 56°43'19.30"W	542	December 2013
18	Santa Fé	21°30'5.32"S, 56°44'37.49"W	485	June 2013 February 2014
19	Ponte Rio Perdido	21°26'59.18"S, 56°47'28.01"W	422	February 2014
20	Ouro Verde	20°43'49.84"S, 56°49'43.98"W	487	March 2011

The species identification was performed with the aid of specialized bibliography (Brown Jr. 1992, Canals 2000, 2003, Casagrande 1995, D’Abrera 1981, 1987a, b, c, 1988, 1994, 1995, Glassberg 2007) and confirmed by specialists (see acknowledgments). The taxonomical classification follows the proposal of Warren et al. (2009) for Hesperiidae, and Lamas (2004) for other families. To confirm new records for the state, we consulted Talbot (1928), Travassos and Freitas (1941), Brown Jr. (1986), Aoki and Sigrist (2006), Rech et al. (2009), Uehara-Prado (2009), Dolibaina et al. (2010), Aoki et al. (2012) and Bogiani et al. (2012).

The effectiveness of the survey was analyzed with individual and sample-based rarefaction curves (Gotelli and Colwell 2001). Sampling effort (by active collection and traps) and the number of individuals and species collected was utilized to obtain the rarefaction curves. All analyses were made with the EstimateS 9.1.0 software (Colwell et al. 2012). Richness was estimated for 80 and 200 random samples for active and trap sampling respectively, using the second order Jackknife estimator. Results are shown as mean ± SD for observed and estimated richness.

## Results and discussion

In total, 768 butterfly individuals were registered, belonging to 146 species in 98 genera, six families in 18 subfamilies (Appendix 1). Nymphalidae was the richest family (82 species), followed by Hesperiidae (22 species), Riodinidae (14 species), Pieridae (12) Papilionidae (11 species) and Lycaenidae (6 species). 239 individuals were sampled in traps, with 48 species of 30 genera, 15 tribes and five subfamilies of Nymphalidae (Table 1). Before this study, 291 species were recorded for MS, from the literature and museum collections (Talbot 1928, Travassos and Freitas 1941, Brown 1986, Aoki and Sigrist 2006, Rech et al. 2009, Uehara-Prado 2009, Dolibaina et al. 2010, Aoki et al. 2012, Bogiani et al. 2012). Here, we provide 37 new records for MS (Appendix 1), summing 328 species for the State.

**Figure 2.**
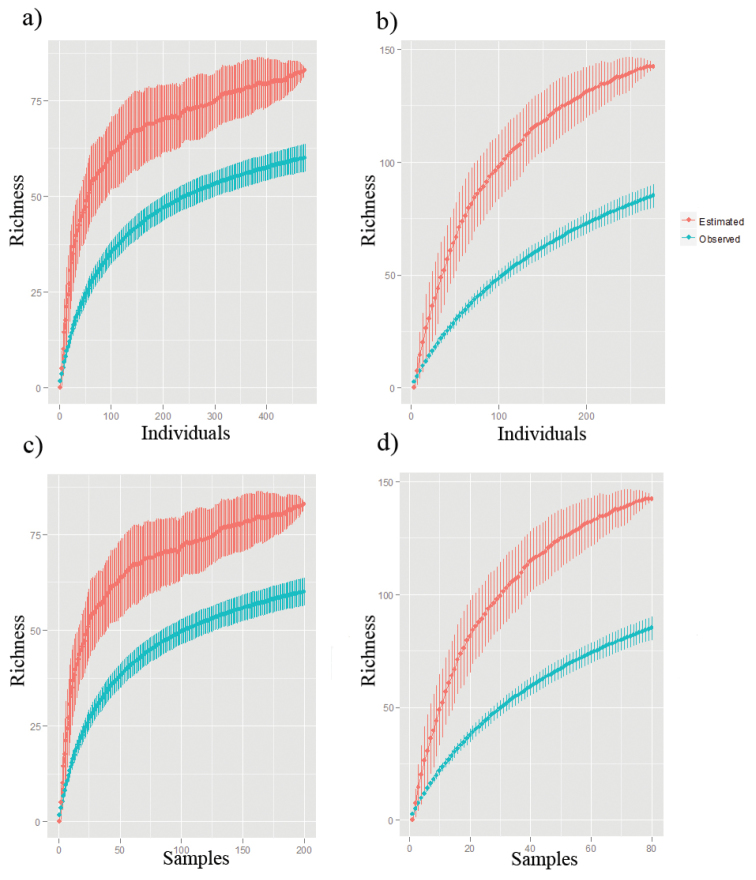
Butterfly richness in the Bodoquena Mountains. Observed and estimated richness of butterflies of the Bodoquena Plateau for both traps (**a, c**) and active collecting (**b, d**), in function of the number of individuals (**a, b**) and sampling effort (**c, d**).

The richest subfamilies were Satyrinae (26 espécies), Biblidinae (24 species), Pyrginae (12 species) and Nymphalinae (10 species). Most new records are represented by rare species with few individuals and low frequency. The most common species were *Eunica macris* (Godart, 1824), *Dynamine artemisia* (Fabricius, 1793) and *Memphis moruus* (Fabricius, 1775). The estimated richness for the Bodoquena Mountains was 83 species for the traps and 142 species for the active collection, while the observed richness was 60 species for the traps and 85 species for the active. Therefore, the results suggest that approximately 72.3% and 59.8% of the species richness of the region were collected with traps and active collection, respectively (Fig. 2). These results indicate that, although traps were more efficient, more species were collected with insect nets. Nevertheless, the rarefaction curves show that the lepidopteran richness in the Bodoquena Plateau may be greater that what we observed in this study.

**Figure 3:**
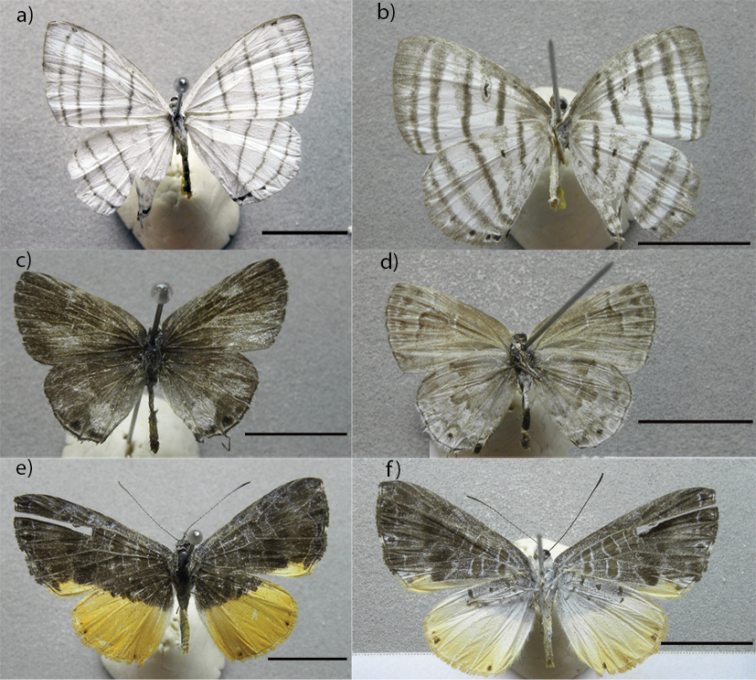
. Rare species collected in the Bodoquena Mountains. *Leucochimona icare* (Hübner, [1819]) (**a, b**), *Strymon mulucha* (Hewitson, 1867) (**c, d**) and *Catocyclotis aemulius* (Fabricius, 1793) (**e, f**). Scale bars …..

Half of the listed species, (52.05% - 76 species) of the butterfly fauna consists of species with records in areas of Cerrado (Brown Jr. and Mielke 1967a, b) and 56.16% (82 species) from the Atlantic Forest (Brown Jr. and Freitas 2000). The vegetation mosaic found in the SBNP can explain this significant number of shared species among biomes. Most species recorded in the Bodoquena Plateau have a wide geographic distribution in Brazil, a fact evidenced in other studies conducted in the Cerrado (Brown Jr. and Mielke 1967a, b). Nevertheless, some rare species were found, such as *Leucochimona icare* (Hübner, [1819]) (Figure 3a, b), *Strymon mulucha* (Hewitson, 1867) (Figure 3d) and *Catocyclotis aemulius* (Fabricius, 1793) (Figure 3e, f). Moreover, two new species of *Moneuptychia* (Nymphalidae) were found and are being described (André V. L. Freitas pers. comm.).

The percentage of 8.27% for Hesperiidae collected in the Bodoquena Plateau, are not in agreement with results of other inventories carried out in the Atlantic Forest (Mielke 1994; Carneiro et al. 2008; Dolibaina et al. 2011), in which hesperiids are usually more common. The Hesperiidae sub-sampling is common in butterflies inventories (Bonfantti et al. 2009; Vasconcelos et al. 2009; Ritter et al. 2011; Zacca and Bravo 2012), especially by their small size, cryptic color patterns and inefficient attraction by fermented fruit lures, thus hindering their collection (Brown Jr. and Freitas 1999; Caldas and Robbins 2003; Zacca and Bravo 2012). In this context, sub-sampling may be derived from different methodologies and approaches of each one of these studies, besides familiarity of the collector with each taxon.

Brown Jr. (1972) discusses and tests the methods used by Ebert (1970) in which the author shows that supplementation of methodologies, proper maintenance of records, performance of several collectors at the same time and knowledge about the behavior of groups guarantee a more complete and representative record of these species. Pinheiro-Machado and Silveira (2006) show that the mentioned method may vary according to the location and logistics, but the best results in number of species are achieved when various methods are employed.

Nymphalidae was the family with highest diversity independent of methodology. This result was expected since this is butterfly family with most species (De Vries 1987), as recorded in the studies by Iserhard and Romanowski (2004), Marchiori and Romanowski (2006), Sackis and Morais (2008), Iserhard et al. (2010) and Rosa et al. (2011) conducted in the state of Rio Grande do Sul. However, Brown Jr. and Freitas (2000) compiled records that resulted in more than 2,100 butterfly species in the Atlantic Forest. According to these authors, in Brazil, the Family Hesperiidae, Nymphalidae and Lycaenidae are the richest in species, followed by Riodinidae, Pieridae, and Papilionidae.

In this study, 32.19% of the species showed were singletons. In the study conducted by Dessuy and Morais (2007) in a forest fragment of Santa Maria, 26% of species collected were singletons, whereas in Sackis (2008) study, it represents 36% of the species. According Dessuy and Morais (2007) singletons are species that live in the habitat in which they are sampled and can be very difficult to find as it keeps in small populations. In addition, these species may be considered rare in one spot, but not in others, due to differences in the availability of food resources, host plants or microclimatic factors (Brown Jr. and Freitas 2003).

The results obtained in this study represent the only information on the butterfly species composition of the Bodoquena Plateau, an area considered of utmost priority for biodiversity conservation. Interestingly, 44.5% of the whole butterfly fauna of MS can be found in the Bodoquena Mountains, showing its great importance for population maintenance and butterfly conservation. Furthermore, the records of rare and new species add evidence to the role of large ecological reserves and conservation areas, as well as the importance of taxonomical surveys. Therefore, this study contributed to the knowledge on Neotropical butterfly species diversity and distribution, providing new records and supporting the use of wildlife inventories as important tools for the knowledge of tropical forests biodiversity and conservation.

## Appendix 1

**Appendix 1.** Butterfly species list for the Serra da Bodoquena National Park, including 20 occurrence sites (represented by codes, see Table 1). Taxa are presented according to family and subfamily. ♦: New records for the Mato Grosso do Sul State; * New species.

Family	Subfamily	Tribe	Species	Abundance	Occurrence site (codes)			
					Bonito	Bodoquena	Jardim	Porto Murtinho
**Nymphalidae (N = 82)**	**Libytheinae**		*Libytheana carinenta* (Cramer, 1777)	1			18	
	**Apaturinae**		*Doxocopa agathina* (Cramer, 1777)	14	4,14,17	13	18	
			*Doxocopa linda nitoris* Fruhstorfer, 1907 ♦	11	4		18	
	**Biblidinae**	**Biblidini**	*Biblis hyperia* (Cramer, 1779)	16	4,14	13	18	
		**Callicorini**	*Callicore pygas* (Godart, [1824])	6	4		11,18	
			*Callicore sorana* (Godart, [1824])	1			11	
			*Haematera pyrame* (Hübner, [1819]) ♦	7	6,9	16	18	
		**Eubagini**	*Dynamine* sp.	4	2,4,9	8		
			*Dynamine aerata* (Butler, 1877) ♦	11	4,9			
			*Dynamine agacles* (Dalman, 1823)	1	9			
			*Dynamine artemisia* (Fabricius, 1793)	50	2,4,9,14,17	3,5,15		
			*Dynamine coenus* (Fabricius, 1793)	1			18	
			*Dynamine postverta* (Cramer, 1779)	9	4,9,14		18	
			*Dynamine postverta postverta* (Cramer, 1779)	9	2,4		18	
		**Catonephelini**	*Eunica macris* (Godart, [1824]) ♦	25	4,6,14	3,5,13,16	18,19	
			*Eunica margarita* (Godart, [1824]) ♦	1	12			
			*Eunica tatila* (Herrich-Schäffer, [1855])	20	4,14		18, 19	
			*Eunica bechina* (Hewitson, 1852)	2	14		18	
		**Ageroniini**	*Hamadryas amphinome* (Linnaeus, 1767)	2			18,19	
			*Hamadryas arete* (Doubleday, 1847)	1	4			
			*Hamadryas chloe* (Stoll, 1787)	1	14	12	18	
			*Hamadryas epinome* (Felder & Felder, 1867)	15	4,9,14	13	11,18	
			*Hamadryas februa* (Hübner, [1823])	21	4,6,12	3,20	11,18	7
			*Hamadryas feronia* (Linnaeus, 1758)	1			11	
			*Hamadryas iphthime* (Bates, 1864) ♦	4	4		10	
		**Epiphelini**	*Nica flavilla* (Godart, [1824])	2	6,12			
			*Temenis laothoe* (Cramer, 1777)	7	1,9	20	10,18,19	
	**Cyrestinae**	**Cyrestini**	*Marpesia chiron* (Fabricius, 1775)	11	4,14,17	3, 16	18	
			*Marpesia petreus* (Cramer, 1776)	1	14			
			*Fountainea ryphea* (Cramer, 1775)	5	12		18	
			*Memphis acidalia* (Hübner, [1819])♦	5	1,2,14	8		
			*Memphis moruus* (Fabricius, 1775)	33	1,4,12,14, 17	8	11,18	
			*Zaretis isidora* (Cramer, 1779)	17	1,4,14	3,130	18,19	
		**Preponini**	*Archaeoprepona demophon* (Linnaeus, 1758)	6	1,2,4,12		18	
			*Prepona pylene* Hewitson, [1854] ♦	1	4			
	**Heliconiinae**	**Heliconiini**	*Heliconius erato phyllis* (Fabricius, 1775)	3	4	16		
			*Dryas iulia* (Fabricius, 1775)	1		16		
			*Dryadula phaetusa* (Linnaeus, 1758)	1		13		
			*Agraulis vanillae maculosa* (Stichel, [1908])	1			11	
	**Danainae**	**Ithomiini**	*Thyridia psidii* Linnaeus, 1758 ♦	1	17			
		**Danaini**	*Lycorea halia pales* Felder & Felder, 1862	2		15		
			*Tithorea harmonia* (Cramer, 1777)	1	6			
	**Limenitidinae**	**Limenitidini**	*Adelpha iphicleola leucates* Fruhstorfer, 1915	6	14,17		18	
			*Adelpha malea* (Felder & Felder, 1861) ♦	1			18	
	**Nymphalinae**	**Nymphalini**	*Colobura dirce* (Linnaeus, 1758)	7	1,2,4,12,14	8		
			*Historis odius* (Fabricius, 1775)	1	4			
			*Smyrna blomfildia* (Fabricius, 1781)	54	4,14		18	
		**Victorinini**	*Siproeta stelenes* (Linnaeus, 1758)	14	4,14		18	
			*Anartia jatrophae* (Linnaeus, 1763)	5	6,12		11,18	
		**Melitaeini**	*Chlosyne lacinia saundersi* (Doubleday, [1847])	5	6	13	11,18	
			*Ortilia ithra* (Kirby, 1900)	5	9,17	5		
			*Ortilia orthia* (Hewitson, 1864) ♦	2	1			
			*Tegosa claudina* (Eschscholtz, 1821)	8	4, 14	3,5,16		
		**Junoniini**	*Junonia evarete* (Cramer, 1779)	2			18	
	**Satyrinae**	**Satyrini**	*Cissia terrestris* (Butler, 1867)	8	1,2,4		18	
			*Hermeuptychia* sp.	3	14		19	
			*Magneuptychia ocnus* (Butler, 1867) ♦	2	2,4			
			*Manataria hercyna* (Hübner, [1821]) ♦	1	14			
			*Moneuptychia* sp.* ♦	6	4,14		18	
			*Moneuptychia* sp.2* ♦	3	14		18	
			*Pareuptychia ocirrhoe* (Fabricius, 1776)	4	2,9		18	
			*Pareuptychia ocirrhoe interjecta* (D’Almeida, 1952)	3	2,12			
			*Pareuptychia summandosa* (Gosse, 1880) ♦	3	1, 14			
			*Paryphthimoides grimon* (Godart, [1824]) ♦	1	14			
			*Paryphthimoides phronius* (Godart, [1824])	9	1,6,9,14	20	18,19	
			*Paryphthimoides poltys* (Prittwitz, 1865)	14	1,4,9,14	20	18,19	
			*Posttaygetis penelea* (Cramer, [1777])	4	1,2,6		18	
			*Taygetina kerea* (Butler, 1869)	6	2	8,15		
			*Taygetis* sp.	5	1,14	8		
			*Taygetis laches* Fabricius, 1793	7	4	8	18,19	
			*Taygetis larua* Felder & Felder, 1867 ♦	2	1			
			*Taygetis mermeria* (Cramer, 1776) ♦	1		15		
			*Taygetis rufomarginata* Staudinger, 1888	1		8		
			*Taygetis sylvia* Bates, 1866 ♦	1		8		
			*Taygetis tripunctata* Weymer, 1907 ♦	1		8		
			*Taygetis virgilia* (Cramer, [1776])	5	4,12		18	
			*Yphthimoides celmis* (Godart, [1824]) ♦	1	9			
		**Brassolini**	*Caligo illioneus* (Cramer, 1775) ♦	2	4		10	
			*Catoblepia berecynthia* (Cramer, 1777) ♦	5	1,2,4	7		
			*Eryphanis reevesii* (Doubleday, [1849]) ♦	3	12		10,18	
			*Opsiphanes invirae* (Hübner, [1808])	8	4,14		18	
		**Morphini**	*Morpho helenor* (Cramer, 1776)	21	4,12	15,20	10,19	
**Papilionidae** **(N = 11)**	**Papilioninae**	**Leptocircini**	*Protesilaus* sp.	1		16		
		**Papilionini**	*Heraclides hectorides* (Esper, 1784) ♦	3		15	18	
			*Heraclides anchisiades* (Esper, 1788)	4	6,9	16		
			*Heraclides isidorus* (Doubleday, 1846) ♦	1	14			
			*Heraclides androgeus* (Cramer, 1775) ♦	2		16		
			*Heraclides astyalus astyalus* (Godart, 1819)	2		16		
			*Heraclides thoas brasiliensis* (Rothschild & Jordan, 1906)	2	1	3		
		**Troidini**	*Battus polydamas polydamas* (Linnaeus, 1758)	1	17			
			*Battus crassus* (Cramer, 1777)	1		16		
			*Parides lysander mattogrossensis* (Talbot, 1928)	2	1,4			
			*Parides neophilus* (Geyer, 1837) ♦	2	1		19	
**Hesperiidae** **(N = 22)**			Hesperiidae sp.	7	1	16		
			Hesperiidae sp. 1	2	1,6			
			Hesperiidae sp. 2	3	1,6	16		
			Hesperiidae sp. 3	4	1,6	16	18	
			Hesperiidae sp. 4	1			18	
			Hesperiidae sp. 5	1			18	
	**Eudaminae**		*Urbanus* sp.	2	4		18	
			*Urbanus dorantes* (Stoll, 1790)	1	6			
			*Urbanus teleus* (Hübner, 1821)	2	6			
	**Hesperiinae**		*Xeniades orchamus orchamus* (Cramer, 1777)	1	14			
	**Pyrginae**	**Erynnini**	*Mylon maimon* (Fabricius, 1775) ♦	1			18	
			*Gorgythion begga begga* (Prittwitz, 1868)	2	1			
		**Pyrgini**	*Xenophanes tryxus* (Stoll, 1780)	2	1,14			
			*Pyrgus orcus* (Stoll, 1780)	7	1		18,19	
			*Pyrgus oileus* (Linnaeus, 1767)	10	1,6			
			*Heliopetes arsalte* (Linnaeus, 1758)	1			18	
			*Heliopetes libra* Evans, 1944	2		16		
			*Heliopetes omrina* (Butler, 1870)	8	1	16	18	
			*Antigonus nearchus* (Latreille, [1817]) ♦	1		3		
			*Antigonus erosus* (Hübner, [1812])	3		16	18	
		**Pyrrhopygini**	*Elbella* sp. ♦	1			19	
			*Myscelus amystis epigona* Herrich-Schäffer, 1869	1			18	
**Riodinidae** **(N = 14)**	**Riodininae**		*Amarynthis meneria* (Cramer, 1776)	1	1			
			*Barbicornis basilis* Godart, [1824]	1	1			
			*Catocyclotis aemulius* (Fabricius, 1793) ♦	1	1			
			*Chalodeta theodora* (Felder & Felder, 1862)	1			18	
			*Chamaelimnas briola meridionalis* (Lathy, 1932) ♦	1	17			
			*Emesis* sp.	6	14	3,16	18	
			*Hyphilaria thasus* (Stoll, 1780)	2			18	
			*Lasaia agesilas agesilas* (Latreille, [1809])	1			18	
			*Leucochimona icare* (Hübner, [1819])	1	1			
			*Notheme erota* (Cramer, 1780)	2		5		
			*Nymphidium leucosia* (Hübner, [1806])	3	1			
			*Rethus periander* (Cramer, 1777)	2		16		
			*Synargis bifasciata* (Mengel,1902) ♦	1		16		
			*Synargis calyce* (Felder & Felder, 1862) ♦	1		16		
**Lycaenidae** **(N = 6)**			*Leptotes cassius* (Cramer, 1775)	5	1,14,17	16		
			*Hemiargus hanno* (Stoll, 1790)	8	6	16	18	
			*Arawacus aetolus* (Sulzer, 1776)	3	17	16		
			*Strymon mulucha* (Hewitson, 1867)	3	1,6		18	
			*Strymon rufofusca* (Hewitson, 1877)	1			18	
			*Strymon ziba* (Hewitson, 1868) ♦	1	14			
**Pieridae** **(N = 12)**	**Coliadinae**		*Eurema* sp.	26	4,6	15,16		
	**Coliadinae**		*Eurema elathea* (Cramer, 1777)	4	14		18	
	**Coliadinae**		*Anteos clorinde* (Godart, [1824])	2		16		
	**Pierinae**	**Pierini**	*Ganyra phaloe endeis* (Godart, 1819) ♦	2		16		
	**Coliadinae**		*Aphrissa statira statira* (Cramer, 1777)	2		16		
	**Coliadinae**		*Itaballia demophile* (Linnaeus, 1763)	6		16		
	**Pierinae**	**Pierini**	*Glutophrissa drusilla* (Cramer, 1777)	4	9	3,16		
	**Coliadinae**		*Phoebis argante* (Fabricius, 1775)	8	6	16		
	**Coliadinae**		*Phoebis sennae* (Linnaeus, 1758)	2		16		
	**Coliadinae**		*Pyrisitia leuce* (Boisduval, 1836)	1		16		
	**Coliadinae**		*Pyrisitia nise* (Cramer, 1775)	3	9	3,5		
	**Coliadinae**		*Rhabdodryas trite* (Linnaeus, 1758)	1		16		
